# Supplementary material for: Erythropoietin N-glycosylation of Therapeutic Formulations Quantified and Characterized: An Interlab Comparability Study of High-Throughput Methods
Source: Biomolecules. 2024 Jan 18;14(1):125. doi: 10.3390/biom14010125 (PMC10813422; doi:10.3390/biom14010125)
Supplement: Supplementary file 1 [file biomolecules-14-00125-s001.zip › biomolecules-2810023-supplementary.pdf]

Supplementary Material

Table of Contents

Table S1: Precision of glycosylation profiling (all analytes).

Table S2: Differences in median CVs (Analytes above 1% relative abundance).

Figure S1: Profile comparability PharmEPO.

Figure S2: Profile comparability Eprex.

Table S3: Fingerprinting resolution.

Table S4: Eprex relative glycan abundances by composition.

The effect of reducing the number of analytes to only those above 1% relative abundance is apparent when comparing Table 1 to Table S1. As expected, all methods show a lower median CV for the abundant analytes than for all analytes.

Table S1. Precision of glycosylation profiling. Descriptive statistics for the distribution of CVs of all analytes.

	Aranesp			PharmEPO			Eprex		
	HA	HP	MS	HA	HP	MS	HA	HP	MS
# of analytes	28	43	68	30	41	76	28	42	76
Median CV<br>(95% CI)	3.4<br>2.3-4.6	2.0<br>1.4-2.5	6.3<br>5.2-7.4	3.0<br>2.4-4.5	1.8<br>1.3-2.6	7.2<br>6.2-8.7	2.1<br>1.5-3.6	2.3<br>1.9-2.9	13.8<br>11.3-16.2
IQR	2.3-4.7	1.1-3.1	3.5-10.0	2.0-4.7	1.2-3.4	4.9-12.2	1.4-3.9	1.5-3.6	7.5-21.5
Min-Max	1.0-5.3	0.5-7.4	1.3-26.1	0.6-11.2	0.4-7.6	1.9-59.8	0.5-10.5	0.3-5.7	1.8-44.2
# < 5% CV	24	40	22	24	37	20	25	39	5

HA = HILIC-FLD(2AB); HP = HILIC-FLD(PROC); MS = MALDI-MS. IQR= interquartile range.

Table S2. Differences in median CVs (Analytes above 1% relative abundance).

p-values	Aranesp	PharmEPO	Eprex
HILIC-FLD(2AB) versus HILIC-FLD(PROC)	**0.0061**	0.1353	0.4855
HILIC-FLD(2AB) versus MALDI-MS	0.2154	**0.0021**	**<0.0001**
HILIC-FLD(PROC) versus MALDI-MS	**<0.0001**	**<0.0001**	**<0.0001**

Discoveries highlighted in bold green.

Differences in median CVs. were tested with a Kruskal-Wallis test with multiple comparsions. Multiple testing correction was applied using a 5% FDR and the Benjamini-Hochberg method. Only analytes above 1% relative abundance were considered.

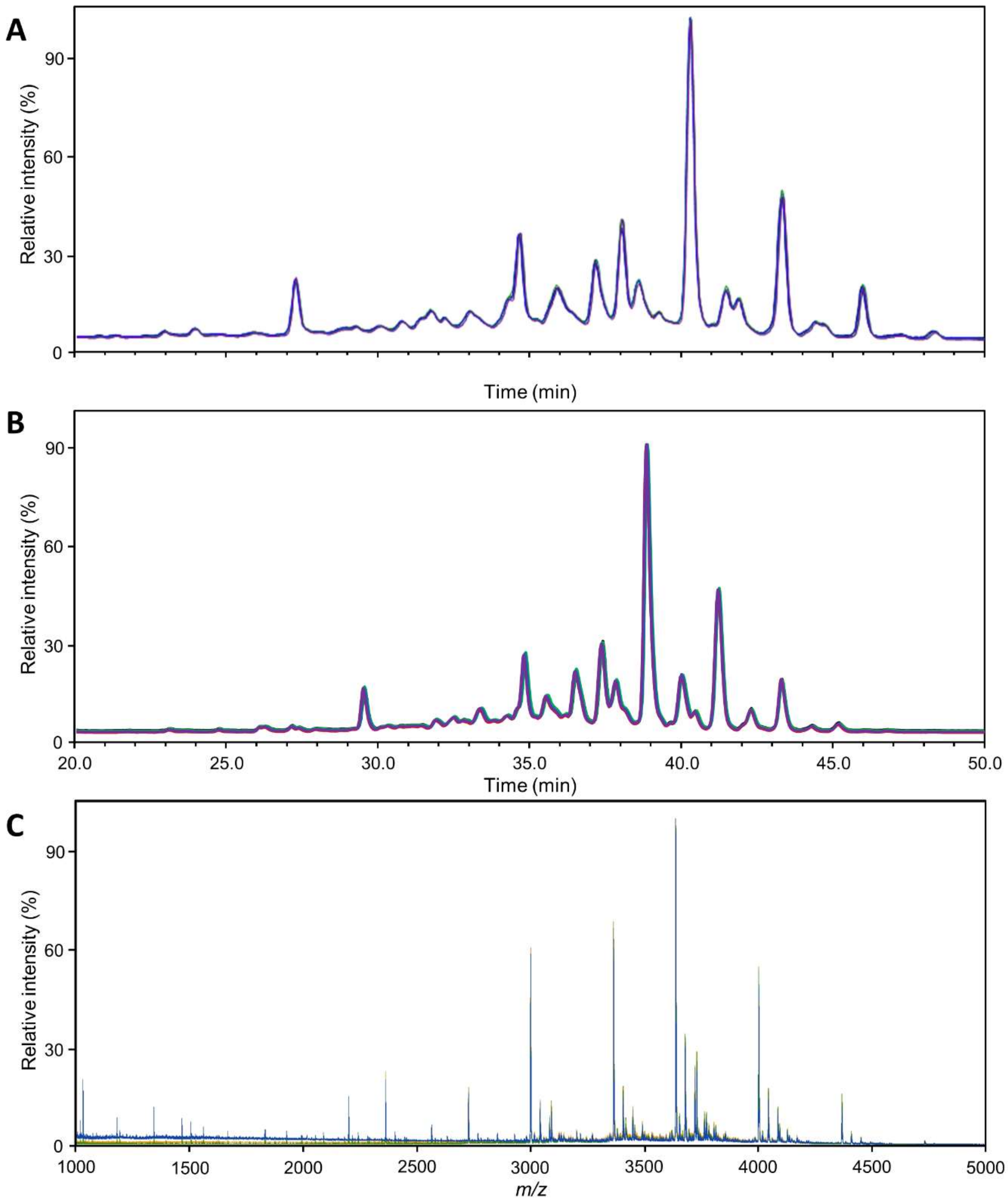

Figure S1. Profile comparability PharmEPO; Overlay of the 5 traces, normalized to the highest peak. A) HILIC-FLD(2AB); B) HILIC-FLD(PROC); C) MALDI-MS.

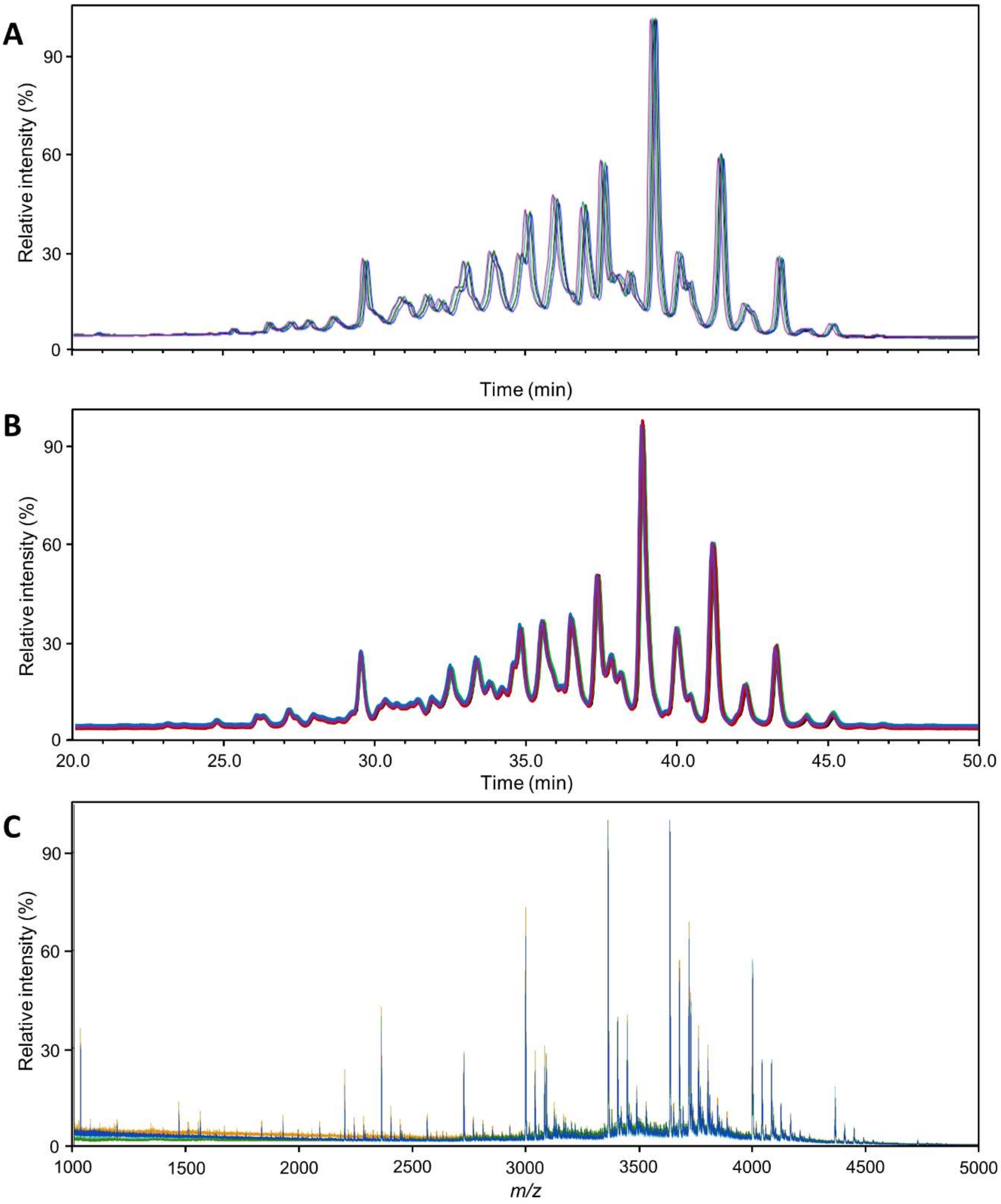

Figure S2. Profile comparability Eprex; Overlay of the 5 traces, normalized to the highest peak. A) HILIC-FLD(2AB); B) HILIC-FLD(PROC); C) MALDI-MS.

Table S3. Fingerprinting resolution: Number of significant* t-tests.

	Aranesp vs PharmEPO	Aranesp vs Eprex	PharmEPO vs Eprex
HILIC-FLD(2AB)	24/25 (96%)	24/25 (96%)	19/25 (76%)
HILIC-FLD(PROC)	31/35 (89%)	33/35 (94%)	27/35 (77%)
MALDI-MS	44/55 (80%)	41/55 (75%)	29/55 (53%)

* α after Bonferroni correction: HILIC-FLD(2AB) $6.7 \cdot 10^{-4}$, HILIC-FLD(PROC) $4.8 \cdot 10^{-4}$, MALDI-MS $3.0 \cdot 10^{-4}$.

Table S4. Eprex relative glycan abundances by composition. KEY α: The number of acetyl (Ac) groups are not specified; the total % contribution is denoted in the structures containing AcX and the number of peaks are highlighted with parenthesis.

Compositions	Structures	MALDI-MS	HILIC-FLD(2AB)/WAX	HILIC-FLD(PROC)-MS
H3N3	**A1**	**nd**	**0.08**	**nd**
H5N2	**Man5**	**nd**	**0.26**	**nd**
**H5N2P1**	**Man5P**	**nd**	**nd**	**0.21**
H3N4	**A2**	**nd**	**0.02**	**nd**
H6N2	**Man6**	**nd**	**0.07**	**nd**
H5N3	**Man5A1**	**nd**	**0.06**	**nd**
**H6N2P1**	**Man6P**	**0.06±0.03**	**nd**	**<0.05**
H3N5	**A3**	**nd**	**0.06**	**nd**
H3N6	**A4**		**0.05**	**nd**
**H3N3F1S1**	***FA1G0S1***	**0.15±0.04**	**nd**	**nd**
H7N2	**Man7**	**nd**	**0.05**	**nd**
**H7N2P1**	***Man7P***	**0.04±0.02**	**nd**	**nd**
H5N4	**A2G2**	**nd**	**0.11**	**nd**
**H4N3F1S1**	***FA1G1S1***	**0.04±0.01**	**0.02**	**nd**
H8N2	**Man8**	**nd**	**0.09**	**nd**
H5N4F1	**FA2G2**	**nd**	**0.20**	**nd**
H5N5	**A3G2**	**nd**	**0.04**	**nd**
H9N2	**Man9**	**nd**	**0.09**	**nd**
**H4N4F1S1**	**FA2[6]G1S1**<br>**FA2[3]G1S1**	**0.20±0.04**	**0.02**<br>**0.03**	**nd**<br>**nd**
**H4N4S2**	***A2G1S2***	**0.07±0.01**	**nd**	**nd**
**H5N4F1S1**	**total**	**0.18±0.03**	**0.50**	**0.35**
H5N4F1S1	FA2G2S1	0.18±0.03	0.45	0.35
H5N4F1S1AcX	FA2G2S1AcX	nd	0.05	nd
**H4N4F1S2**	**total**	**1.22±0.29**	**nd**	**nd**
H4N4F1S2	*FA2G1S2*	0.76±0.16	nd	nd
H4N4F1S2AcX	*FA2G1S2AcX*	0.46±0.13	nd	nd
H4N4F1S2Ac1	*FA2G1S2Ac1*	0.24±0.06	nd	nd
H4N4F1S2Ac2	*FA2G1S2Ac2*	0.23±0.07	nd	nd
**H5N4S2**	***A2G2S2***	**0.10±0.02**	**nd**	**nd**
**H5N5F1S1**	***FA2G1L1S1***	**0.09±0.01**	**0.02**	**nd**
**H5N4F1S2**	**total**	**2.60±0.38**	**2.74**	**3.65**
H5N4F1S2	FA2G2S2	1.90±0.25	2.05	3.05
H5N4F1S2AcX	FA2G2S2AcX	0.71±0.14	0.69(2)	0.60
H5N4F1S2Ac1	FA2G2S2Ac1	0.44±0.09	α	0.34
H5N4F1S2Ac2	FA2G2S2Ac2	0.27±0.06	α	0.26
**H5N4F1S1Gc1**	**total**	**0.15±0.03**	*****	*****
H5N4F1S1Gc1	*FA2G2S2 (1xNeuAc+1xNeuGc)*	0.08±0.02	*	*
H5N4F1S1Gc1Ac1	*FA2G2S2Ac1*	0.08±0.01	*	*
**H6N5F1S1**	**total**	**0.12±0.01**	**0.20**	**nd**
H6N5F1S1	FA3G3S1	0.12±0.01	0.16(2)	nd
H6N5F1S1AcX	FA3G3S1AcX	nd	0.04	nd
**H5N5F1S2**	**total**	**0.60±0.08**	**nd**	**nd**
H5N5F1S2	FA3G2S2	0.40±0.05	nd	nd
H5N5F1S2AcX	FA3G2S2AcX	0.20±0.04	nd	nd

H5N5F1S2Ac1	FA3G2S2Ac1	0.12±0.02	nd	nd
H5N5F1S2Ac2	FA3G2S2Ac2	0.09±0.02	nd	nd
**H5N5F1S1Gc1**	***FA3G2S2<br>(1xNeuAc+1xNeuGc)***	**0.09±0.01**	*	*
H6N6F1S1	**FA4G3S1**	**nd**	**0.02**	**nd**
**H6N5F1S2**	**Total**	**2.39±0.14**	**1.70**	**1.16**
H6N5F1S2	FA2G2L1S2<br>FA3G3S2	1.64±0.08	0.04<br>1.48	0.85
H6N5F1S2AcX	FA3G3S2AcX	0.71±0.08	0.18(1)	0.31
H6N5F1S2Ac1		0.39±0.04	α	nd
H6N5F1S2Ac2	FA3G3S2Ac2	0.32±0.04	α	0.31
H7N6F1S1	**FA4G4S1**	**nd**	**0.13**	**nd**
**H5N5F1S2Gc1**	***FA3G2S3<br>(2xNeuAc+1xNeuGc)***	**0.23±0.03**	*	*
**H6N6F1S2**	**FA3G2L1S2**	**0.26±0.01**	**0.04**	**nd**
**H6N5F1S3**	**Total**	**9.32±0.90****	**5.10****	**4.2****
H6N5F1S3	FA3G3S3	4.89±0.27	5.10(2)	2.03
H6N5F1S3AcX	FA3G3S3AcX	4.43±0.64	nd	2.17
H6N5F1S3Ac1	FA3G3S3Ac1	1.68±0.19	nd	1.68
H6N5F1S3Ac2	FA3G3S3Ac2	1.72±0.26	nd	0.49
H6N5F1S3Ac3		0.66±0.11	nd	nd
H6N5F1S3Ac4		0.36±0.08	nd	nd
**H6N5F1S2Gc1**	**Total**	**0.56±0.06**	*	*
H6N5F1S2Gc1	*FA3G3S3<br>(2xNeuAc+1xNeuGc)*	0.27±0.04	*	*
H6N5F1S2Gc1Ac1		0.29±0.03	*	*
**H7N6F1S2**	**Total**	**2.72±0.29**	**2.15**	**2.17**
H7N6F1S2	FA4G4S2<br>FA3G3L1S2<br>FA2G2L2S2	1.85±0.23	1.19<br>0.42<br>0.02	nd
H7N6F1S2AcX	FA4G4S2AcX	0.87±0.07	0.52(1)	nd
H7N6F1S2Ac1		0.51±0.03	α	nd
H7N6F1S2Ac2		0.36±0.03	α	nd
H8N7F1S1	**FA3G3L2S1<br>FA4G4L1S1**	**nd**	**0.02<br>0.05**	**nd**
**H6N6F1S3**	**Total**	**0.60±0.03**	**2.19**	**nd**
H6N6F1S3	FA4G3S3	0.32±0.02	1.96	nd
H6N6F1S3AcX	FA4G3S3AcX	0.28±0.02	0.23(4)	nd
H6N6F1S3Ac1		0.28±0.02	α	nd
**H6N6F1S2Gc1**	*FA4G3S3<br>(2xNeuAc+1xNeuGc)*	**0.27±0.02**	*	*
**H7N6F1S3**	**Total**	**17.10±0.61**	**13.80**	**18.66**
H7N6F1S3	FA4G4S3	9.39±0.56	7.69	9.14
H7N6F1S3AcX	FA4G4S3AcX<br>FA3G3L1S3AcX	7.71±0.31	5.22(7)<br>0.89	9.52
H7N6F1S3Ac1	FA4G4S3Ac1	3.44±0.13	α	4.98
H7N6F1S3Ac2	FA4G4S3Ac2	3.42±0.17	α	2.37
H7N6F1S3Ac3	FA4G4S3Ac3	nd	α	1.42
H7N6F1S3Ac4	FA4G4S3Ac4	0.85±0.09	α	0.75
**H7N6F1S2Gc1**	*FA4G4S3<br>(2xNeuAc+1xNeuGc)*	**0.46±0.11**	*	*
H8N7F1S2	**Total**	**nd**	**1.32**	**nd**
H8N7F1S2	FA4G4L1S2	nd	0.86	nd

	FA3G3L2S2		0.22	
H8N7F1S2AcX	FA4G4L1S2AcX	nd	0.22(1)	nd
**H9N8F1S1**	**FA4G4L2S1**	**nd**	**0.02**	**nd**
H7N7F1S3	**Total**	**nd**	**2.35**	**nd**
H7N7F1S3AcX	FA4G3L1S3AcX	nd	2.35	nd
**H7N6F1S4**	**Total**	**29.54±1.77**	**43.79**	**39.63**
H7N6F1S4	FA4G4S4	10.79±0.18	11.78	9.94
H7N6F1S4AcX	FA4G4S4AcX	18.75±1.87	31.99(7)	29.69
H7N6F1S4Ac1	FA4G4S4Ac1	5.63±0.23	α	7.5 (3)
H7N6F1S4Ac2	FA4G4S4Ac2	6.64±0.60	α	9.09 (3)
H7N6F1S4Ac3	FA4G4S4Ac3	3.17±0.44	α	4.4
H7N6F1S4Ac4	FA4G4S4Ac4	2.39±0.42	α	4.31 (2)
H7N6F1S4Ac5	FA4G4S4Ac5	0.92±0.20	α	2.12
H7N6F1S4Ac6	FA4G4S4Ac6	nd	α	1.08
H7N6F1S4Ac7	FA4G4S4Ac7	nd	α	0.74
H7N6F1S4Ac8	FA4G4S4Ac8	nd	α	0.45
**H7N6F1S3Gc1**	**Total**	**1.63±0.16**	*	*
H7N6F1S3Gc1	*FA4G4S4 (3xNeuAc+1xNeuGc)*	0.75±0.12	*	*
H7N6F1S3Gc1Ac1		0.88±0.05	*	*
**H8N7F1S3**	**Total**	**7.75±0.80**	**4.16**	**7.00**
H8N7F1S3	FA3G3L2S3<br>FA4G4L1S3	4.44±0.60	0.84<br>3.32	7.00
H8N7F1S3Ac1		1.63±0.13	nd	nd
H8N7F1S3Ac2		1.16±0.07	nd	nd
H8N7F1S3Ac3		0.53±0.02	nd	nd
H9N8F1S2	**FA4G4L2S2**	**nd**	**0.24**	**nd**
H8N8F1S3	**FA4G3L2S3**	**nd**	**0.38**	**nd**
**H8N7F1S4**	**Total**	**15.23±1.08**	**9.59**	**16.08**
H8N7F1S4	FA4G4L1S4	6.45±0.73	7.17	8.25
H8N7F1S4AcX	FA4G4L1S4AcX	8.78±0.41	2.42(1)	7.83
H8N7F1S4Ac1	FA4G4L1S4Ac1	3.07±0.25	α	4.26
H8N7F1S4Ac2	FA4G4L1S4Ac2	3.05±0.15	α	3.57 (2)
H8N7F1S4Ac3		1.39±0.06	α	nd
H8N7F1S4Ac4		0.90±0.06	α	nd
H8N7F1S4Ac5		0.37±0.03	α	nd
**H8N7F1S3Gc1**	**Total**	**0.83±0.15**	*	*
H8N7F1S3Gc1	*FA4G4L1S4 (3xNeuAc+1xNeuGc)*	0.39±0.09	*	*
H8N7F1S3Gc1Ac1		0.44±0.05	*	*
**H9N8F1S3**	**Total**	**1.34±0.30**	**1.40**	**2.15**
H9N8F1S3	FA4G4L2S3	0.97±0.24	1.40	2.15
H9N8F1S3AcX	FA4G4L2S3AcX	0.37±0.06	nd	nd
H9N8F1S3Ac1		0.37±0.06	nd	nd
H10N9F1S2	**FA4G4L3S2**	**nd**	**0.04**	**nd**
**H9N8F1S4**	**Total**	**3.78±0.68**	**3.81**	**3.15**
H9N8F1S4	FA4G4L2S4	1.89±0.43	2.80	3.15
H9N8F1S4AcX	FA4G4L2S4AcX	1.89±0.25	1.01(1)	nd
H9N8F1S4Ac1	FA4G4L2S4Ac1	0.78±0.14	α	nd
H9N8F1S4Ac2	FA4G4L2S4Ac2	0.62±0.08	α	nd
H9N8F1S4Ac3	FA4G4L2S4Ac3	0.26±0.02	α	nd
H9N8F1S4Ac4	FA4G4L2S4Ac4	0.16±0.01	α	nd
H9N8F1S4Ac5	FA4G4L2S4Ac5	0.07±<0.01	α	nd

**H10N9F1S3**	**FA4G4L3S3**	**nd**	**0.21**	**0.52**
**H10N9F1S4**	**Total**	**0.28±0.06**	**1.47**	**0.52**
H10N9F1S4	FA4G4L3S4	0.17±0.05	1.21	0.52
H10N9F1S4AcX	FA4G4L3S4AcX	0.11±0.02	0.27	nd
H10N9F1S4Ac1	FA4G4L3S4Ac1	0.07±0.01	α	nd
H10N9F1S4Ac2	FA4G4L3S4Ac2	0.05±0.01	α	nd
**H11N10S3AcX**	FA4G4L4S3AcX	**nd**	**0.07**	**nd**
**unidentified**				0.55

#HILIC–FLD(2AB)/WAX does not distinguish the number of O-acetylations present

*This/these methods do not differentiate NeuAc and NeuGc

**The abundance of H6N5F1S3/FA3G3S3 is far lower in the HILIC methods than in MALDI-MS. For the HILIC-FLD(2AB)/WAX this is easily explained by the lack of detection of the O-acetylated variants. However, for HILIC-FLD(PROC)-MS the abundance is lower in all related species.

nd: not detected .
